# Constitutive p53 heightens mitochondrial apoptotic priming and favors cell death induction by BH3 mimetic inhibitors of BCL-xL

**DOI:** 10.1038/cddis.2015.400

**Published:** 2016-02-04

**Authors:** J Le Pen, M Laurent, K Sarosiek, C Vuillier, F Gautier, S Montessuit, J C Martinou, A Letaï, F Braun, P P Juin

**Affiliations:** 1 UMR 892 INSERM/6299 CNRS/Université de Nantes, Team 8 ‘Cell Survival And Tumor Escape In Breast Cancer', Institut de Recherche en Santé de l'Université de Nantes, Nantes, France; 2 Dana-Farber Cancer Institute, Harvard Medical School, Boston, MA, USA; 3 Institut de Cancérologie de l'Ouest, Centre de Lutte contre le Cancer René Gauducheau, Saint Herblain, France; 4 Department of Cell Biology, University of Geneva, Geneva,Switzerland

## Abstract

Proapoptotic molecules directly targeting the BCL-2 family network are promising anticancer therapeutics, but an understanding of the cellular stress signals that render them effective is still elusive. We show here that the tumor suppressor p53, at least in part by transcription independent mechanisms, contributes to cell death induction and full activation of BAX by BH3 mimetic inhibitors of BCL-xL. In addition to mildly facilitating the ability of compounds to derepress BAX from BCL-xL, p53 also provides a death signal downstream of anti-apoptotic proteins inhibition. This death signal cooperates with BH3-induced activation of BAX and it is independent from PUMA, as enhanced p53 can substitute for PUMA to promote BAX activation in response to BH3 mimetics. The acute sensitivity of mitochondrial priming to p53 revealed here is likely to be critical for the clinical use of BH3 mimetics.

Major tumor suppressors pathways, such as these relying on pRB and/or protein 53 (p53), promote proapoptotic signals that ultimately converge on Mitochondrial Outer Membrane Permeabilization (MOMP).^1^ As a consequence, their loss in cancer cells results in failure to undergo MOMP in response to therapy, and approaches allowing to mitigate such defects are being actively investigated.

The BCL-2 (B-cell lymphoma/leukemia-2) family proteins are key regulators of MOMP and subsequent apoptosis.^2, 3, 4^ They are subdivided into three groups depending on their BCL-2 homology (BH) domain composition and their function: the multidomain anti-apoptotic proteins (BCL-2-like 1 (BCL-xl), BCL-2 and myeloid cell leukemia-1 (MCL-1), the multidomain proapoptotic proteins (BCL-2-associated X protein (BAX), BCL-2 antagonist/killer-1 (BAK)) and the BH3-only pro-apoptotic members (BCL-2-associated death promoter (BAD), Bcl-2-interacting mediator of cell death (BIM), BH3-interacting-domain death agonist (BID), NOXA and p53-upregulated modulator of apoptosis (PUMA)).^5, 6, 7^ Cell-fate decisions triggered by apoptotic stimuli are based on the relative amount of each BCL-2 protein as well as on the interplay between members of this family.^5, 8, 9^ One proximal step is the conversion of inert monomeric molecules of BAX/BAK into dimers that nucleate higher order oligomerization and lead to mitochondrial damage.^10, 11, 12^ This process of ‘activation' can be induced by a subset of BH3-only proteins that directly interact with BAX/BAK (the so-called activators, BIM, BID and PUMA). Conversely, antiapoptotic proteins prevent this by interacting with BAX/BAK and/or activators.^13, 14^ This relies on the binding of the BH3 domain of the proapoptotic proteins to a hydrophobic cleft formed by the BH1-2 and -3 domains of BCL-2 homologs.^15^ This can now be pharmacologically modulated by ‘BH3-mimetics' that target more or less selectively the BH3-binding pockets of BCL-2, BCL-xL or MCL-1.^16^

BH3 mimetics directly promote MOMP by releasing BH-3 activators and BAX/BAK from antiapoptotic proteins, hence their use may help restore apoptosis in cancer cells harboring defects in tumor suppressor pathways. However, tumor suppressors may provide additional cooperating signals that foster BH3 mimetic induced cell death, and whose absence may reciprocally limit BH3 mimetics efficiency. Consistent with the latter view, we recently showed that the pRB/E2F-1 pathway amplifies cell death induced by BCL-2/BCL-xL inhibition, by mediating caspase-dependent induction of the endogenous MCL-1 inhibitor NOXA.^17^ Likewise, p53, as a transcription factor, was shown to induce the expression of various apoptotic BCL-2 family genes^18, 19^ in addition to directly interacting with some BCL-2 family proteins.^20, 21, 22, 23, 24, 25, 26, 27, 28^ So far, no comprehensive study has investigated which, if any, of these effects may be critical to BH3-mimetic induction of cell death. We herein show that p53, even when expressed in viable, dividing cancer cells, promotes death signals that critically cooperate with BH3 mimetic treatment to trigger cell death.

## Results

### Constitutive expression of p53 in HCT116 p21−/− cells contributes to induction of cell death by the BCL-2/BCL-xL inhibitor ABT-737

**Figure 1:**
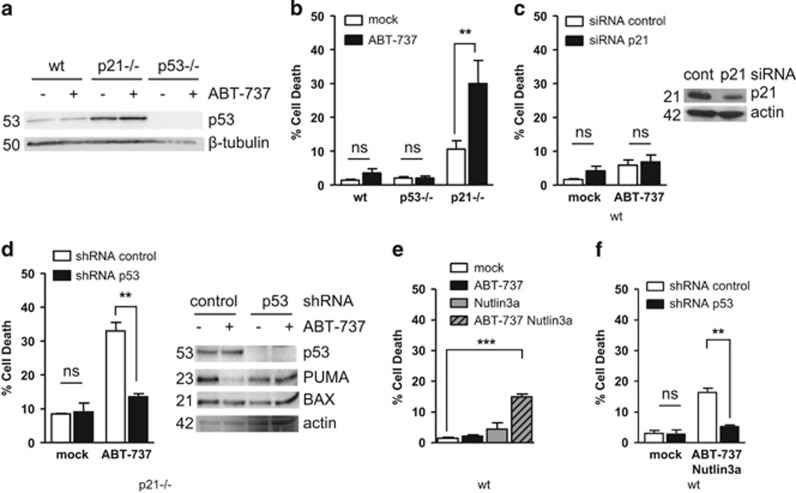
p53 is involved in sensitivity to ABT-737. (**a**) HCT116 wt, p53−/− or p21−/− cells were treated for 24h by 2*μ*M ABT-737 for western-blot analysis of p53 expression. (**b**) Cells were treated as in **a** before cell death measurement. (**c**) HCT116 wt cells were transfected with control or p21 siRNAs. Forty-eight hours later, cells were treated by 2 *μ*M ABT-737 for 24 h before western blot (inset) and cell death measurement. (**d**) HCT116 p21−/− cells previously infected with control or p53 shRNAs were treated by 2 *μ*M ABT-737 for 24 h before cell death analysis and western blot. (**e**) HCT116 wt were treated for 24 h by 2 *μ*M ABT-737 and/or 10 *μ*M Nutlin3a before cell death analysis (one-way ANOVA and Bonferroni post test). (**f**) HCT116 wt cells previously infected with a control or p53 shRNAs were treated by 2 *μ*M ABT-737 and 10 *μ*M Nultin3a for 24 h before cell death analysis. Cell death was assessed by a trypan blue staining procedure. Corresponding molecular weight on western blots are indicated (kDa). Data presented are mean±S.E.M. of three independent experiments. **P<0.01, ***P<0.001

We have previously established that the colorectal cancer HCT116 p21−/− cell line is a model cell line that requires sustained inhibition of PUMA and BAX by BCL-xL to survive. This cell line is therefore a useful model to study the mechanisms leading to BAX-dependent cell death following BH3 mimetic inhibition of BCL-xL.^13^ Independently from p21 loss, the HCT116 p21−/− cells were shown to express constitutively high levels of p53^29^ (see also Figure 1a).

These cells are more sensitive than the parental (HCT116 wt) cell line to treatment with the BH3 mimetic inhibitor of BCL-2 and BCL-xL, ABT-737 (Figure 1b). Whereas down regulation of p21 by RNA interference in wild-type cells has no detectable impact on their resistance to ABT-737 (Figure 1c), downregulation of p53 in p21 null cells by a lentiviral-based shRNA significantly decreased their sensitivity (Figure 1d). Reciprocally, treatment of HCT116 wt cells with the Mouse double minute 2 homolog (Mdm2) inhibitor Nutlin-3a enhanced p53 expression and the response to ABT-737 (Figure 1e) whereas HCT116 p53−/− cells remained resistant to the combined treatment (data not shown). This effect was inhibited by down regulation of p53 (Figure 1f). Of note, p21 levels were induced by Nutlin-3a treatment in these assays, further underscoring the inconsequence of p21 expression on sensitivity to ABT-737 (Supplementary Figure S1A). Similar results were observed in the breast cancer Cal51 cell lines where ABT-737 and Nutlin-3a treatment synergized to induce p53-dependent cell death (Supplementary Figure S1B–C).

Altogether, these results show that p53, when expressed above a certain threshold, contributes to the apoptotic response induced by ABT-737.

### p53 does not sensitize cells to ABT-737 through transcriptional induction of NOXA, PUMA or BAX

**Figure 2:**
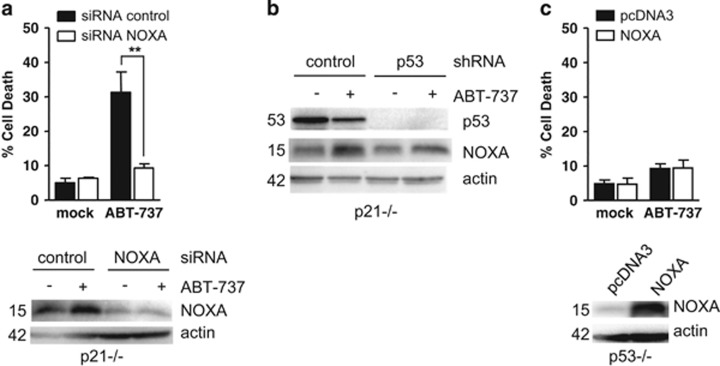
p53-independant induction of NOXA is involved in HCT116 p21−/− sensitivity to ABT-737. (**a**) HCT116 p21−/− cells were transfected by control or NOXA siRNAs for 48 h and treated for 24 additional hours by 2 *μ*M ABT-737 before cell death analysis and western blot. (**b**) HCT116 p21−/− cells previously infected with a control or p53 shRNAs were treated by 2 *μ*M ABT-737 for 24h before western blot analysis. (**c**) HCT116 p53−/− cells were transfected with pcDNA3 or pcDNA3-NOXA vector. Twenty-four hours later, cells were treated by 2 *μ*M ABT-737 for 24 h before cell death analysis. Data presented are mean±S.E.M. of three independent experiments. **P<0.01

PUMA and BAX are major actors of the apoptotic response of HCT116 p21−/− cells to ABT-737, whereas BIM did not appear to contribute to these responses.^13^ Moreover, silencing of NOXA expression prevented cell death induced by ABT-737 (Figure 2a), indicating that this endogenous MCL-1 inhibitor has a role in cell death induction by BCL-2/BCL-xL inhibition. As NOXA, PUMA and BAX are transcriptional targets of activated p53,^30^ we investigated whether their constitutive induction by p53 prior treatment, and/or their induction by p53 during treatment, had a role.

NOXA expression was increased in HCT116 p21−/− upon ABT-737 treatment (Figure 2a), as reported in other cell lines.^17, 31^ Such induction was still detectable in p53-depleted cells (Figure 2b) and overexpression of NOXA in HCT116 p53-null cells neither induced cell death by itself nor sensitized them to ABT-737 treatment (Figure 2c). This argues that NOXA is an inducible actor of ABT-737-triggered apoptosis. However its induction does not absolutely require p53 and it is insufficient *per se*, in the absence of p53, to induce efficient cell death in combination with ABT-737.

**Figure 3:**
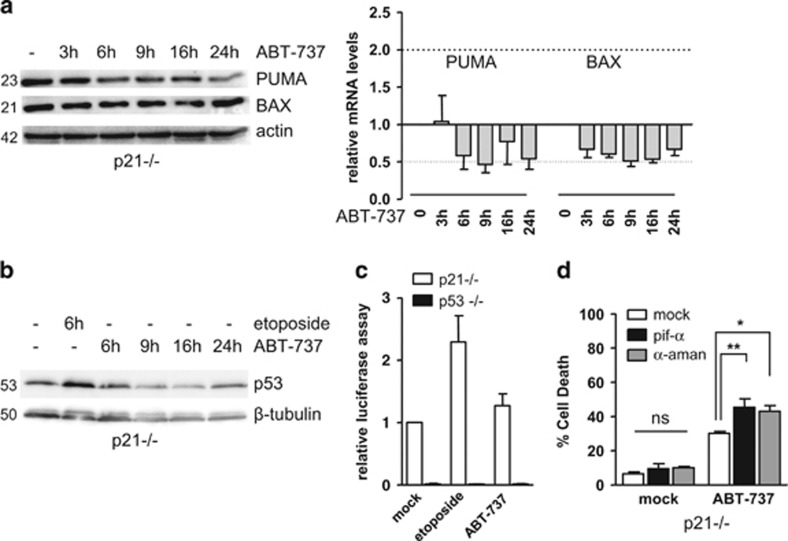
p53 transcriptionnal activity is dispensable for cell death induction by ABT-737. (**a** and **b**) HCT116 p21−/− cells were treated for the indicated time by 2 *μ*M ABT-737 or 20 *μ*M Etoposide (a genotoxic agent used as a positive control for p53 activation) before western blot analysis and qPCR. (**c**) HCT116 p21−/− and p53−/− transfected with TA-luciferase control vector or p53-reporter luciferase vector, in parallel to a pCMV-*β*-galactosidase vector as transfection control. Twenty-four hours later, cells were treated for amanitin (w/o e) 6 h by 2 *μ*M ABT-737 or 50 *μ*M Etoposide, before lysis and luciferase assay. (**d**) HCT116 p21−/− were pretreated 24 h by 40 *μ*M Pifithrin-*α* (pif- *α*) or 10 *μ*M *α*-Amanitin (*α*-Aman), before treatment for 24 h by 2 *μ*M ABT-737 and cell death analysis. Data presented are mean±S.E.M. of three independent experiments. *P<0.05, **P<0.01

The situation was different for PUMA and BAX. Indeed, investigation of kinetic changes in PUMA and BAX protein and mRNA expression levels during ABT-737 treatment brought no support to the hypothesis that their expression was induced at all (and *a fortiori* by p53) during treatment (Figure 3a). Moreover, PUMA and BAX were not detectably affected by silencing of p53 in HCT116 p21−/− cells, whether these were untreated or treated 24 h with ABT-737 (Figure 1d).

Indeed, ABT-737 had no effect on the expression of p53 itself in the p21−/− cells (Figure 3b) and the transcriptional activity of p53, as measured in a luciferase reporter assay, was not detectably modified by ABT-737 treatment in p21-null cells (Figure 3c). Importantly, neither Pifitrin-*α* (an inhibitor of p53-dependent transcriptional activation), nor *α*-Amanitin (an inhibitor of RNA polymerase-mediated transcription) inhibited cell death induced by ABT-737 in HCT116 p21−/− cells (Figure 3d) arguing that the transcriptional activity of p53 is dispensable for cell death induction by ABT-737 in these cells. Notably, we even noted a stimulatory effect of these compounds. This evokes a preceding publication who ascribed this enhancement to the fact that p53 transcriptional activity is not only dispensable for apoptotic responses, but also a restrain to the execution of p53 mitochondrial death program.^32^ Pretreatment with Pifitrin-*α* of the wild-type cells did not decrease cell death induced by the combined ABT-737 and Nutlin-3a treatment, indicating that the transcriptional activity of p53 is dispensable under these conditions (Supplementary Figure S1D).

**Figure 4:**
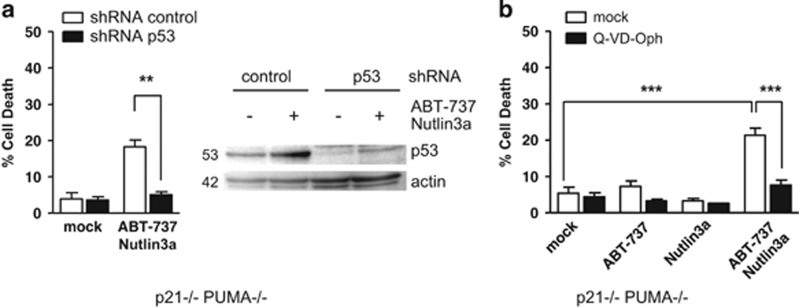
p53 favors sensitivity to ABT-737 in the absence of PUMA. (**a**) HCT116 p21−/− PUMA−/− cells were infected with control or p53 shRNAs before treatment for 24 h with 2 *μ*M ABT-737 and 10 *μ*M Nutlin3a and western blot and cell death analysis. (**b**) HCT116 p21−/− PUMA−/− cells were treated for 24 h by 2 *μ*M ABT-737, 10 *μ*M Nutlin3a and/or 10 *μ*M of the pan caspase inhibitor Q-VD-OPh, before cell death analysis. Data presented are mean±S.E.M. of three independent experiments. **P<0.01, ***P<0.001

Finally, it should be noted that the effects exerted by p53 are not functionally equivalent to those of the BH3-only proteins NOXA and/or PUMA. As shown below, p53 exerts apoptotic effects even in cells depleted of MCL-1 and thus they cannot solely rely on the induction of NOXA (which essentially functions as an inhibitor of MCL-1) or of NOXA-like molecules. Moreover, we found that in HCT116 p21−/− PUMA−/− cells, enhancement of p53 expression by Nutlin-3a treatment sensitized these otherwise resistant cells to ABT-737 treatment (Figure 4a and b). Cell death induced under these conditions was inhibited by silencing of p53 by RNA interference (Figure 4a) and by pretreatment with a broad-spectrum caspase inhibitor Q-VD-OPh (Figure 4b), whereas pretreatment with Pifitrin-*α* did not decrease the level of cell death (Supplementary Figure S1D).

Our data support the notion that p53 exerts an effect that adds to these of NOXA and PUMA to promote cell death in ABT-737 cells, and that this effect is in great part independent of its transcriptional activity.

### p53 favors BAX activation in ABT-737-treated cells

As BAX has a major role in cell death induced by ABT-737 treatment or BCL-xL loss in HCT116 p21−/− cells,^13^ we investigated the influence of p53 on BAX ‘activation' by ABT-737 treatment.

**Figure 5:**
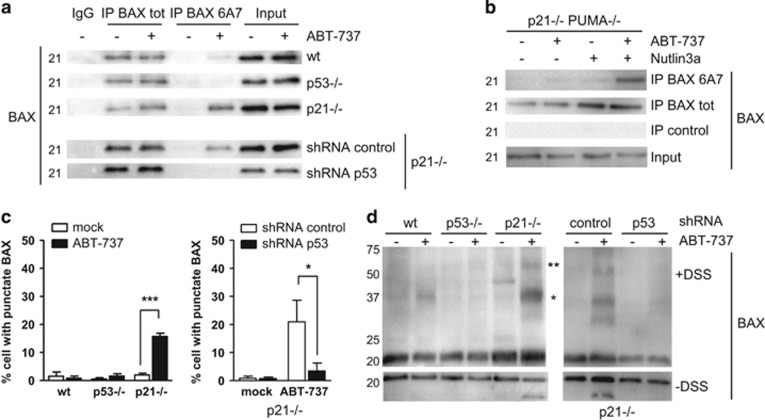
p53 favors BAX activation upon ABT-737 treatment. (**a**) HCT116 wt, p53−/−, p21−/− or p21−/− previously infected with control or p53 shRNAs were treated for 16h by 2*μ*M ABT-737, before immunoprecipitation using a conformation specific-BAX antibody (6A7) and a non-conformation specific BAX antibody (BAX tot.) (**b**) HCT116 p21−/− PUMA−/− were treated for 20 h by 2 *μ*M ABT-737 and/or 10 *μ*M Nutlin3a, before immunoprecipitation using the indicated anti-BAX antibodies. (**c**) HCT116 wt, p53−/−, p21−/−, or p21−/− previously infected with control or p53 shRNAs were treated for 20 h by 2 *μ*M ABT-737. Cells were stained using Mitotracker Red, and a BAX-antibody. More than 50 cells were observed per treatment and cells exhibiting punctate BAX staining were counted. (**d**) HCT116 wt, p53−/−, p21−/−, or p21−/− previously infected with control or p53 shRNAs were treated for 16 h by 2 *μ*M ABT-737. Hundred micrograms of proteins were crosslinked by DSS before western blot analysis. (*) (**): sizes in agreement with BAX oligomers. Data presented are mean±S.E.M. of three independent experiments. *P<0.05, **P<0.01 and ***P<0.001

We first evaluated conformation changes in the amino-terminus domain of BAX by immunoprecipitation assays with the 6A7 antibody. 6A7 immunoreactive forms of BAX were detected in ABT-737-treated p21-null cells, but only weakly in wild-type cells and not in p53-null cells, indicating that the insensitivity of the wild-type and p53-null cells coincides with an absence of efficient activation of BAX (Figure 5a). The induction of the 6A7 positive form was severely impaired in p21-null cells depleted in p53 arguing that p53 favors conformation change amplitudes of BAX in response to ABT-737 (Figure 5a). Importantly, even though PUMA is a major driver of BAX ‘activation' in ABT-737 treated HCT116 p21−/− cells,^13^ a significant conformation change in BAX was detected in HCT116 p21−/−PUMA−/− cells treated by the combination ABT-737/Nutlin3a (Figure 5b). This implies that enhanced p53 can substitute for PUMA to promote BAX activation and subsequent cell death (as shown in Figure 4).

Other features of BAX activation are mitochondrial translocation and oligomerization. To evaluate BAX mitochondrial translocation, percentages of cells with punctate BAX distribution were determined after ABT-737 treatment (Figure 5c and Supplementary Figure S2). BAX showed a punctate staining pattern that colocalized with a mitochondrial marker upon ABT-737 treatment in p21-null cells but not in cells infected with a p53 shRNA lentivector (Figure 5c). No translocation of BAX was observed in the wild-type cells and in p53-null cells, consistent with the absence of 6A7-positive BAX molecules in these cells (Figure 5c). In agreement with data above, oligomerization of BAX, assessed by crosslinking with disuccinimidyl suberate (DSS) was detected in ABT-737-treated p21-null cells, but only weakly in wild-type cells and not in p53-null cells. This oligomerization of BAX in ABT-737 treated HCT116 p21-null cells was abolished by silencing of p53 (Figure 5d).

### p53 modulates both the inhibition of BCL-xL by ABT-737 and death signals downstream of BCL-xL

BAX activation is critically kept in check by the BH3-binding activity of BCL-xL in HCT116 p21−/− cells, as shown by the fact that inhibition of BCL-2/BCL-xL by ABT-737, and BCL-xL down regulation, both trigger BAX-dependent cell death.^13^ In further support to this, treatment of these cells with WEHI-539, a selective BCL-xL inhibitor, triggered p53-dependent cell death (Supplementary Figure S3). This indicates that p53 acts by favoring derepression of BCL-xL sensitive death signals by BH3 mimetics and/or by inducing death signals once BCL-xL is inhibited.

**Figure 6:**
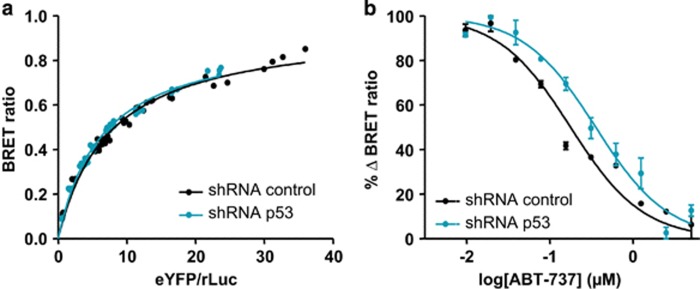
Effect of p53 on BCL-xL binding to BAX. (**a**) Donor saturation assay analysis: saturation curves were performed in HCT116 p21−/− cells previously infected with control (black) or p53 shRNAs (blue) using increasing amount of vectors encoding YFP-BCL-xL in the presence of a fixed amount of the vector-encoding Rluc-BAX. BRET ratios (BRET Unit) were measured for every YFP-BCL-xL plasmid concentrations and are plotted as a function of the ratio of total acceptor to donor fluorescence (YFP–BCL-xL/Rluc-BAX). The data were fitted using a nonlinear regression equation assuming a single binding site. (**b**) Dose–response effect of ABT-737 on BCL-xL/BAX BRET signals. HCT116 p21−/− cells previously infected with control (black) or p53 (blue) shRNAs were transiently transfected with a constant amount of plasmid DNA encoding the donor Rluc-BAX (50 ng) and plasmid DNA encoding the acceptor YFP-BCL-xL (200 ng), treated or not with increasing doses of ABT-737 (from 0.1 to 10 *μ*M). %ΔBRET is plotted as a function of the logarithmic ABT-737 concentrations. The data were fitted using a nonlinear regression. Data shown represent results from one of three separate BRET titration experiments (performed in duplicate) that produced qualitatively similar results

To investigate the former, we measured the spacial proximity between transiently transfected Luciferase-fused BAX and YFP-fused BCL-xL by bioluminescence resonance energy transfer (BRET) in live cells expressing p53 or not, treated or not with ABT-737. As previously reported,^33^ saturable BRET signals were observed between donor BAX and increasing levels of acceptor BCL-xL, indicative of a specific interaction between these proteins. Specificity was further confirmed by assessing that BRET signals were significanly less intense when a variant of YFP-BCL-xL carrying a single point-mutation in the BH3 binding site was used (Supplementary Figure S4). The saturation curves that were observed between BAX and wild-type BCL-xL in control HCT116 p21−/− and in p53-depleted cells were almost identical, suggesting that constitutive p53 does not impact on the propensity of BCL-xL to sequester BAX (Figure 6a). We then investigated the influence p53 might have on the sensitivity of these interactions to ABT-737. The effect of a range of ABT-737 concentrations on BAX/BCL-xL BRET signals in control and p53-deleted cells was evaluated. Silencing of p53 in p21-null cells consistently enhanced the resistance of BAX/BCL-xL interactions (evaluated by BRET) to ABT-737 (Figure 6b), indicating that constitutive p53 sensitizes BAX/BCL-xL interactions to ABT-737. Of note, p53 is not absolutely required for ABT-737 to inhibit BAX/BCL-xL interactions, as comparable inhibitions of BRET signals by concentrations above 1 *μ*M of ABT-737 were measured in p53-proficient and p53-deficient cells. This may explain why we detected no difference in the effects of ABT-737 (used at 2 *μ*M) on endogenous BAX/BCL-xL when we grossly evaluated them by co-immunoprecipitation assays in lysates from treated wild-type and p21−/− HCT116 cells (Supplementary Figure S5).

**Figure 7:**
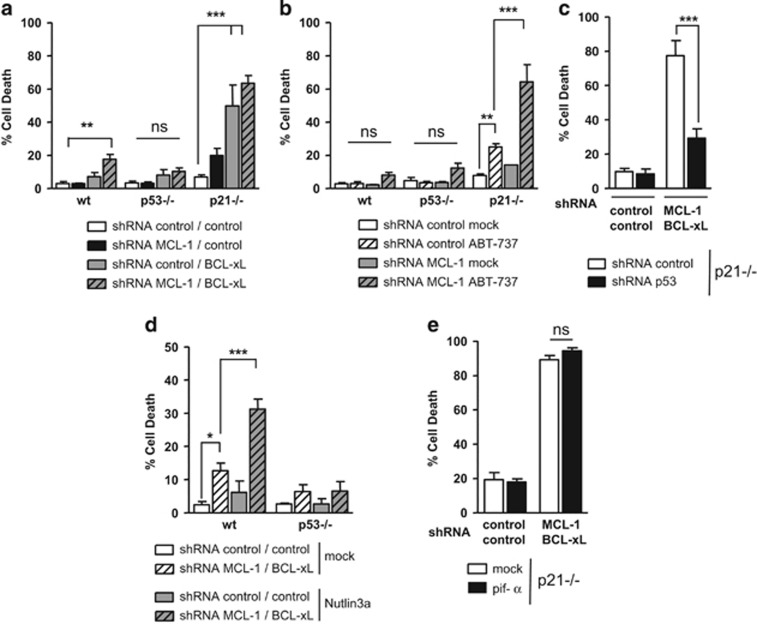
p53 contributes to efficient cell death induced by silencing of MCL-1 and BCL-xL. (**a**) HCT116 wt, p53−/− or p21−/− cells were infected with control or MCL-1 shRNAs. Six hours later, cells were infected with control or BCL-xL shRNAs. Twenty-four hours later, cells were washed, and cell death was assessed after forty-eight additional hours. (**b**) HCT116 wt, p53−/− or p21−/− cells were infected with control or MCL-1 shRNAs. Cells were washed after 24 h, and treated 16 h by 2 *μ*M ABT-737 before cell death analysis. (**c**) HCT116 p21−/− cells previously infected with control or p53 shRNAs were infected with control or MCL-1 shRNAs. Six hours later, cells were infected with control or BCL-xL shRNAs. Twenty-four hours later, cells were washed, and cell death was assessed after 48 additionnal hours. (**d**) HCT116 wt or p53−/− cells were infected with control or MCL-1 shRNAs. Six hours later, cells were infected with control or BCL-xL shRNAs. Twenty-four hours later, cells were washed and treated by 10 *μ*M Nutlin3a for 48 h before cell death analysis. (**e**) HCT116 p21−/− cells were infected as in **c** and 24 h later, cells were washed and treated by 40 *μ*M Pifithrin-*α* (pif-*α*). Cell death was assessed after 48 additional hours. Data presented are mean±S.E.M. of three independent experiments. *P<0.05, **P<0.01 and ***P<0.001

To evaluate whether p53 might exert an apoptotic effect beyond its modulation of BCL-xL sensitivity to ABT-737, we analyzed whether it would have a role even in cells deleted in BCL-xL. In agreement with previous results,^13^ silencing of BCL-xL in p21-null cells promoted a more efficient cell death in itself than ABT-737 treatment. This effect was not significantly enhanced by silencing of MCL-1 (Figure 7a), whereas silencing of MCL-1 strongly increased cell death of p21−/− cells treated with ABT-737 (Figure 7b). Silencing of the two antiapoptotic proteins lead to a weak level of cell death of HCT116 wt cells (Figure 7a) that was enhanced by Nutlin3a treatment (Figure 7d). Little cell death was measured in the p53 null cells in all these conditions (Figure 7a and c). Moreover, Pifithrin-*α* did not inhibit cell death in response to the silencing of BCL-xL and MCL-1 in p21 null cells (Figure 7e). Thus, p53 is required to promote cell death in cells in which the proapoptotic actors are no more sequestered by the anti-apoptotic counterparts, independently from its transcriptional activity.

As a whole, these data indicate that, in order to favor BAX activation and cell death in response to BH3 mimetics, p53 both facilitates derepression of BAX from BCL-xL by the BH3 mimetics and full-blown BAX activation once BCL-xL is inhibited (or it expression down regulated).

### p53 heightens mitochondrial apoptotic priming

**Figure 8:**
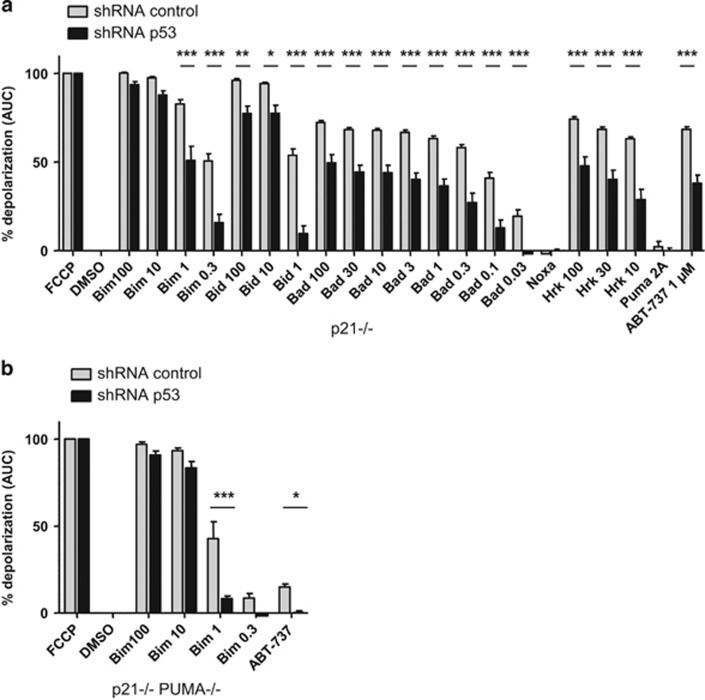
p53 impacts on mitochondrial priming. Mitochondrial depolarization of digitonin-permeabilized HCT116 p21−/− cells (**a**) or HCT116 p21−/− PUMA−/− cells (**b**) previously infected with control or p53 shRNAs, stained with the mitochondrial potential sensitive JC1 dye, and treated with a panel of BH3 peptides. Percent depolarization is shown as the area under the curve (AUC) normalized to positive control fully depolarized mitochondria (FCCP). Dimethyl sulfoxide (DMSO) serves as the negative control. Data presented are mean±S.E.M. of three independent experiments. *P<0.05, **P<0.01 and ***P<0.001

We inferred from the above results that, in addition to having a specific effect on the response of BAX/BCL-xL interactions to BH3 mimetics, constitutive p53 would exert a promiscuous effect that amplifies mitochondrial damage in response to most (if not all) perturbations of the BCL-2 network. To test this in an integrated manner, we performed BH3 profiling assays. In these assays, we evaluated, as a marker of mitochondrial integrity, the mitochondrial potential of permeabilized HCT116 p21−/− or p21−/− PUMA−/− cells (in which p53 was downregulated or not) incubated with a range of BH3 peptides and with ABT-737. As shown in Figure 8a, HCT116 p21−/− cells expressing PUMA and p53 (that is, infected with a control shRNA) showed dose-dependent response to BAX activating peptides (BIM-BH3 and BID-BH3) and the sensitizing peptides BAD-BH3 (which targets BCL-2/BCl-xL) and Harakiri (HRK)-BH3 (which only targets BCL-xL) but did not respond to the NOXA-BH3 peptide (which inhibits MCL-1). This is in agreement with a critical role for BCL-xL in maintaining the survival of these apoptosis competent cells. Downregulation of p53 by shRNA decreased the response to active peptides. Whereas p53-depleted cells had a diminished response even to the highest concentrations of BAD-BH3 and HRK-BH3, they only resisted to intermediate concentrations of BID and BIM-BH3 peptides (Figure 8a). This indicates that constitutive p53 may provide an active mitochondrial damaging signal that remains limiting when sensitizing peptides are used but that can be bypassed by high concentrations of activating peptides. Moreover, BH3 profiling experiments were consistent with the notion that this signal is independent from an effect of PUMA on mitochondrial priming (ref. 13 and manuscript in preparation). PUMA-deficient cells had, indeed, diminished responses to low doses of BIM-BH3 and to ABT-737 (compare Figure 8b with 8a), that were further decreased by p53 depletion (Figure 8b). Altogether, these data indicate that constitutive p53 heightens mitochondrial priming by an effect that is independent from, and adds to, that of PUMA.

## Discussion

ABT-737 and its orally available equivalent ABT-263 do not target MCL-1 but inhibit both BCL-2 and BCL-xL. As the latter is arguably the most potent anti-apoptotic protein of the family and a major contributor to drug resistance, such compounds are likely to have a role to play in a chemotherapeutic setting. The clinical use of BH3 mimetic inhibitors of BCL-xL is, however, limited by their secondary effects, in particular on platelets. Identifying parameters that determine the therapeutic window of these compounds is therefore important. It is thus of particular relevance to report that p53, even when expressed constitutively under conditions where it does not influence the expression of its proapoptotic transcription targets, enhances cell death induced by BCL-xL inhibition. On one hand, this implies that BH3 mimetics may not totally substitute for the lack of an active p53 tumor suppressor in cancer cells. On the other hand, it implies that healthy tissues may be more harmed than anticipated when BCL-xL inhibitors are combined with chemotherapeutic agents that even mildly affect p53.

BH3 mimetics, because they only inhibit selective subsets of antiapoptotic proteins, efficiently induce apoptosis when two types of signals are also present: signals that inhibit other anti-apoptotic proteins (MCL-1 in the case of ABT-737) and signals that trigger BAX activation. Our results are in line with preceding results and underscore that, to the very least in the case of ABT-737 treatment of the cells used here, NOXA contributes to the former signals, and constitutive PUMA to the latter. However, p53 does not promote ABT-737 induction of cell death solely via expression of either one of these two main actors. Indeed, p53 exerts a proapoptotic activity even in MCL-1-depleted cells. Thus, the apoptotic function of p53 extends beyond inhibition of MCL-1 by induction of NOXA. Moreover, p53 was dispensable for ABT-737 treatment to induce expression of NOXA, an effect we recently reported to rely on the pRB/E2F-1 pathway in p53 mutant cells.^17^ We also found no evidence of a role of p53 in the expression of PUMA in cells where it nevertheless contributes to cell death. The fact that enhancement of p53 expression sensitizes PUMA knock out cells to ABT-737 demonstrates that p53 exerts a BAX-dependent apoptotic effect that is independent from PUMA, even though it might cooperate with it to favor cell death.

That constitutive p53 heightens mitochondrial priming in cooperation with (and not via) BH3-only proteins is particularly well illustrated by BH3 profiling assays. These assays reveal that down regulation of p53 impacts on the mitochondrial response to numerous BH3 peptides, even in cells where this response is lowered by PUMA knock out (which impacts in itself on mitochondrial priming independently from p53). p53 depletion diminishes the response to low concentrations of activator BH3 peptides but not to higher concentrations. This is mostly consistent with the notion that constitutive p53 provides signals that cooperate with BH3-mediated activation of BAX, and that are dispensable for MOMP to occur when a sufficient amount of activator BH3 molecules are present. In agreement with this, we found that p53 was required for BAX translocation, change of conformation and oligomerization to be fully patent in cells treated with BH3 mimetics, even though all these events can be reproduced in the absence of p53 in cell-free assays.^5, 13^

Another important implication from the BH3 profiling assays is that the effect of p53 on MOMP lies, at least partly, downstream of anti-apoptotic proteins, as p53 depletion confers resistance to induction of MOMP by the highest concentrations of the sensitizer BAD and HRK-BH3 peptides we could test. In further support of this idea, we found that p53 still had an apoptotic role in cells that were depleted in BCL-xL and/or MCL-1. This implies that p53 would exert a promiscuous, positive, effect on BAX activity regardless of the identity of the antiapoptotic proteins that keep this activity in check. Our observations may thus extend to BCL-2 or MCL-1 inhibition. Arguably, the effect of p53 on the sensitivity of BCL-xL/BAX interactions to ABT-737 might appear as an additional effect, as it implies that p53 might have a specific influence on BCL-xL. We cannot formally rule out, however, that it ensues from a single effect on BAX that favors its displacement from BCL-xL in the same time as its activation.

Pretreatments with either Pifithrin-*α* (or *α*-Amanitin) did not inhibit cell death induced by ABT-737, or by the silencing of MCL-1 and BCL-xL, which is reminiscent of their lack of inhibitory effect on Nutlin-induced p53-dependent apoptosis in HCT116 cells.^32, 34^ This argues that p53 would exert at least some of its proapoptotic effects by a transcription-independent function,^21, 23, 24, 26, 27, 35, 36, 37, 38^ possibly from the cytoplasm where we found p53 to partly reside in the cells used here (data not shown). It is particularly relevant here to recall that the DNA binding domain of p53 can interact with BCL-xL. This interaction is favored by tetramerization of p53 and it influences the BH3 binding activity of BCL-xL even though it does not occur at the BH3 binding site *stricto sensu*.^21, 23, 24^ p53 might also directly activate BAX and or BAK by a similar, yet to be fully characterized, type of interaction.^27, 28, 36, 37^ A recombinant p53 constructs encompassing residues 79–393^38^ had no detectable effect on the priming of HCT116 p21−/− cells (whether they had been depleted in p53 by RNA interference or not prior the assays, data not shown). When incubated with mitochondria isolated from BAX/BAK null mouse embryonic fibroblasts, this recombinant p53 did not trigger cytochrome c release by itself, did not activate recombinant BAX, did not enhance the effects of tBID on BAX, and did not derepress the inhibitory effects of BCL-xL (Supplementary Figure S6). Since this construct lacks the N-terminal transcriptional activation region, this brings support to the notion that this latter portion, but not the DNA binding domain *per se* is involved in BAX activation.^39, 40^ *Cis*–*trans* interconversion of Pro47 in this domain by the prolyl isomerase Pin1 was shown recently to enhance p53 induced BAX activation.^41^ Of note, we could not find evidence of specific interactions between constitutive p53 and either BAX or BCL-xL in co-immunoprecipitations assays in the cells used in this study, and we failed to detect specific BRET signals between ectopic p53 and any of these BCL-2 proteins (data not shown). We thus can not formally rule out that p53, in addition to its direct effects on BAX, might exert other effects on mitochondrial apoptotic priming.

Taken together, our data unravel widespread effects of p53 on mitochondrial priming. Mitochondrial priming may thus acutely respond to the many stresses that stabilize and activate p53. As BH3 profiling assays are quite sensitive to detect such changes, that can occur without any overt activation of a p53 transcriptional program, this underscores their potential as biomarkers predicting cytotoxicity (and BCL-2 dependency) in a dynamic manner^42^ and the role of mitochondria as a «sounding board» of stress signals. From a clinical standpoint, the preferred sensitivity to BH3 mimetics of cells that express higher levels of p53 implies that BH3 mimetic treatment may skew clonal diversity and counter-select for cells with less (or less responsive) p53. Understanding the mechanistic basis for p53 induced priming, and finding approaches to bypass the consequences of p53 defects on this priming, are therefore required to avoid this bias.

## Material and methods

### Cell culture

The Cal51 cell line obtained from ATCC, was grown in RPMI medium. The HCT116 cell lines (parental, p21−/−, p53−/− and p21−/−PUMA−/−), grown in McCoy's 5A, were kindly provided by Dr. B. Vogelstein (John Hopkins Kimmel Cancer Center, Baltimore, MD, USA). When specified, we used ABT-737 at 2 *μ*M, WEHI-539 at 2 *μ*M, Nutlin-3a at 10 *μ*M (Sigma, St. Louis, MO, USA), Etoposide at 50 *μ*M (Sigma), Pifithrin-*α* at 40 *μ*M (Sigma), *α*-Amanitin at 10 *μ*M (Sigma), QVD-OPh at 10 *μ*M (R&D System, Minneapolis, MN, USA).

### Plasmids, RNA interferences and recombinant proteins

Cells were transfected with pcDNA3 (as a control vector) or pcDNA3-NOXA vector using Lipofectamine2000 (Invitrogen, Carlsbad, CA, USA). Control siRNA or p21 siRNA (Santa-cruz Biotechnologies, Santa Cruz, CA, USA, respectively #44230 and #29427) were transfected using HiPerfect transfection reagent (Qiagen, Valencia, CA, USA). We used the following lentivirus: PFG12 and PLKO1; bacterial LacZ as control (5′-gtgaccagcgaatacctgt-3′), human BCL-xL (5′-AGGATACAGCTGGAGTCAG-3′), and human MCL-1 (5′-gaatgccagtgacctgtgt-3′); p53 and control were from Addgene, Cambridge, MA, USA (respectively #25636 and #8453). Lentivirus were engineered, produced and titrated as previously described.^13^ A multiplicity of infection of three was used, and further experiments were performed 2 days after infection. Cells infected by PLKO1 were selected by adding 1 *μ*g/ml of puromycine to the medium, and cells were used during a maximum of 4 weeks.

The recombinant His-tagged full length BAX and caspase-8-cleaved BID (tBID) were prepared as previously described.^43^ The recombinant p53 was kindly provided by Professor Halazonetis and prepared as previously described.^38^

### Cellular assays

Bioluminescence Resonance Energy Transfert assays were performed as previously described.^33^ Briefly, the indicated cells were seeded on 12 plates and transfected with 50 ng/well of pRLuc-BAX vector (BRET donor, BAX fused to luciferase), and increasing amount (for saturation curves), or 200 ng/well (for dose-response curves), of peYFP-BCL-xL vector (BRET acceptor, BCL-xL fused to eYFP). Twenty-four hours later, cells were reseeded into white 96-well plate and treated as indicated. Light emissions at 485 and 530 nm were measured consecutively by using the Mithras fluorescence-luminescence detector LB-940 (Berthold, Thoiry, France) after addition of 5 *μ*M of the luciferase substrate (coelenterazine H from Uptima, MontLucon, France). The BRET signal was determined by calculating the ratio of the light emitted by the acceptor protein eYFP-BCL-xL (530 nm) over the light emitted by the donor protein Rluc-BAX (475 nm), and corrected by subtracting the background signal detected with a non-tagged BCL-xL.

Mitochondrial profiling was performed as previously described.^44^ Briefly, 15 *μ*l of BH3 peptide diluted in T-EB (300 mM Trehalose, 10 mM HEPES-KOH pH7.7, 80 mM KCl, 1 mM EGTA, 1 mM EDTA, 0.1% BSA, 5 mM succinate) were deposited per well in a black 384-well plate. Cells were washed in T-EB, resuspended and added to one volume of a dye solution containing 4 *μ*M JC-1, 40 *μ*g/ml oligomycin, 0.02% digitonin, 20 mM 2-mercaptoethanol in T-EB. This cell/dye solution stood 10 min at RT to allow permeabilization and dye equilibration. Fifteen microliters of the cell/dye mix was added to each treatment well of the plate (final concentration of 2 × 10^4^ cells/well), shaken for 15 s inside the reader, and the fluorescence at 590 nm monitored every 5 min at 32 °C for 3 h. Percentage loss of Ψ*μ* for the peptides is calculated by normalization to the solvent only control DMSO and the positive control FCCP. PUMA2A is an inert double alanine-substituted PUMA BH3 peptide serving as a negative control. Individual DBP analysis were performed using triplicates for DMSO, FCCP, and the different BH3 concentrations used, and the expressed values stand for the average of three different readings.

Luciferase assays were performed by cotransfecting cells with TA-luciferase control vector or p53-reporter luciferase vector (Affymetrix, Santa Clara, CA, USA, #LR0000 and #LR0057, respectively) and with pCMV-*β*-galactosidase vector. After treatment, cells were lysed under agitation in PLB (Promega, Madison, WI, USA) and luciferase activity was measured using Dual-Luciferase Reporter Assay System (Promega). *β*-galactosidase activity was measured using the specific assay reporter system (Promega). The luciferase activity was reported to the *β*-galactosidase activity, and is shown relative to the untreated condition.

Coimmunoprecipitation assays, using 1 *μ*g of the indicated antibody and 500 *μ*g of protein lysates in 1% CHAPS buffer with protease phosphatase inhibitors, were performed with protein A/G-agarose PureProteome system (Millipore) as previously described.^13^ CHAPS buffer has been previously reported not to activate BAX.^45, 46^ After western blotting, proteins were revealed using clean-blot IP reagent Pierce (Thermo Fisher Scientific, Rockford, IL, USA).

For immunocytochemical assays, cells were incubated 15 min with 150 nM de MitoTracker Red CMXRos (Invitrogen), washed with PBS and fixed with 1% paraformaldehyde in PBS for 30 min at RT. Cells were washed in PBS and permeabilized with 0.1% SDS in PBS for 10 min at RT. Cells were washed and, after a 30-min saturation with 5% BSA in PBS at 37 °C, were incubated with primary antibodies diluted in 1% BSA in PBS overnight at 4 °C (BAX A3533, Dako, Glostrup, Denmark). After three washes in PBS, incubation with secondary antibodies (anti-rabbit IgG coupled to Alexa Fluor 488) was performed in 1% BSA in PBS for 2 h at 37 °C. Cells were then washed and mounted in ProLong Gold antifade reagent (Invitrogen). Representative images were acquired on Zeiss Axiovert 200M.

After washing with PBS, cells for crosslinking experiments were lysed on ice using 1% CHAPS buffer with protease phosphatase inhibitors, and extract were cleared by centrifugation 15 min 13 000 g at 4 °C. Hundred micrograms of protein extract were incubated with 5 mM of DSS in Hepes 20 mM buffer. After 30 min agitation at room temperature, 50 mM of Tris-HCl pH7.5 was added to stop the reaction. After 15 min of incubation, crosslinked extracts were denaturated for electrophoresis and western-blotting.

Cell viability was determined by a trypan blue staining procedure.

### Immunoblot and antibodies

After two washings with cold PBS, cells were lysed in ice-cold lysis buffer (SDS 1% EDTA 10 mM; Tris-HCl pH8,1 50 mM; protease inhibitors, Na3VO4 1 mM, NaF 100 × ) and extracts were sonicated. Protein extracts were separated by SDS-PAGE, transferred onto a PVDF membrane (Millipore, Billerica, MA, USA) and revealed with a chemiluminescence kit (Millipore). Presented Western-Blot are representative of three independent experiments. Following antibodies were used: actin (MAB1501R, Millipore), *β*-tubulin (T0198, Sigma), BAX (A3533, Dako), BCL-xL (1018-1, Epitomics), cytochrome c (BD-Pharmingen San Diego, CA, USA), MCL-1 (sc-819, Santa Cruz Biotechnologies, Santa Cruz, CA, USA), NOXA (ALX-804-408, Enzo Life Science, New York, NY, USA), PUMA (12450, Cell Signalling), p21 (2947, Cell Signalling), p53 (#554294, BD-Pharmigen). The above antibodies against BAX, BCL-xL and p53 were also used for immunoprecipitations, as those against BAX 6A7 (ab5714, Abcam, Cambridge, UK) and FLAG-tag (F1804, Sigma).

### Quantitative PCR

Total RNA were extracted from cells using a kit Nucleospin RNA (Macherey-Nagel, Düren, Germany) following the manufacturer instructions. Retrotranscription was permormed from 500 ng RNA using Maxima First strand cDNA Synthesis kit for RT-q-PCR (Fisher Scientifique, Pittsburgh, PA, USA). Quantitative PCR was performed with EuroBioGreen qPCR Mix Lo-ROX (Eurobio, Courtaboeuf, France) from 4 ng of retrotranscripted RNA using a Stratagene Mx3005P (Agilent, Santa Clara, CA, USA). Data were analyzed using Pfaffl method with three references genes (*ActB, RPLP0 and RPS18*)^47^ and presented in mRNA levels relative to untreated cells.

Following primers were used:

BAX (5′-GCAACTTCAACTGGGGCCGGG/GATCCAGCCCAACAGCCGCTC-3′);

PUMA (5′-GGAACAGTGGGCCCGGGAGA/GTGCCGCTGCTGCTCCTCTT-3′);

ActB (5′-AGAAAATCTGGCACCACACC/CAGAGGCGTACAGGGATAGC-3′);

RPLP0 (5′-AACCCAGCTCTGGAGAAACT/CCCCTGGAGATTTTAGTGGT-3′);

RPS18 (5′-ATCCCTGAAAAGTTCCAGCA/CCCTCTTGGTGAGGTCAATG-3′).

### Data analysis

Data were from three independent experiments. Unless otherwise stated, statistical analysis of data was performed using two-ways ANOVA with Bonferonni post-test on GraphPad Prism (La Jolla, CA, USA). Error bars represent S.E.Ms. The following symbols are used: *, ** and *** that correspond to a *P*-value inferior to 0.05, 0.01 and 0.001, respectively.

## Abbreviations

BAD: BCL-2-associated death promoter
BAK: BCL-2 antagonist/killer-1
BAX: BCL-2-associated X protein
BCL-2: B-cell CLL/lymphoma 2
BCL-xL: BCL-2-like 1
BH: BCL-2 homology
BID: BH3-interacting-domain death agonist
BIM: Bcl-2-interacting mediator of cell death
BRET: bioluminescence resonance energy transfer
HRK: Harakiri
MCL-1: myeloid cell leukemia-1
MOMP: mitochondrial outer membrane permeabilization
Mdm2: Mouse double minute 2 homolog
p53: protein 53
PUMA: p53-upregulated modulator of apoptosis
