# Supplementary material for: Constitutive p53 heightens mitochondrial apoptotic priming and favors cell death induction by BH3 mimetic inhibitors of BCL-xL
Source: Cell Death Dis. 2016 Feb 4;7(2):e2083–. doi: 10.1038/cddis.2015.400 (PMC4849148; doi:10.1038/cddis.2015.400)
Supplement: Supplementary Figures [file cddis2015400x1.ppt]

## Slide 1
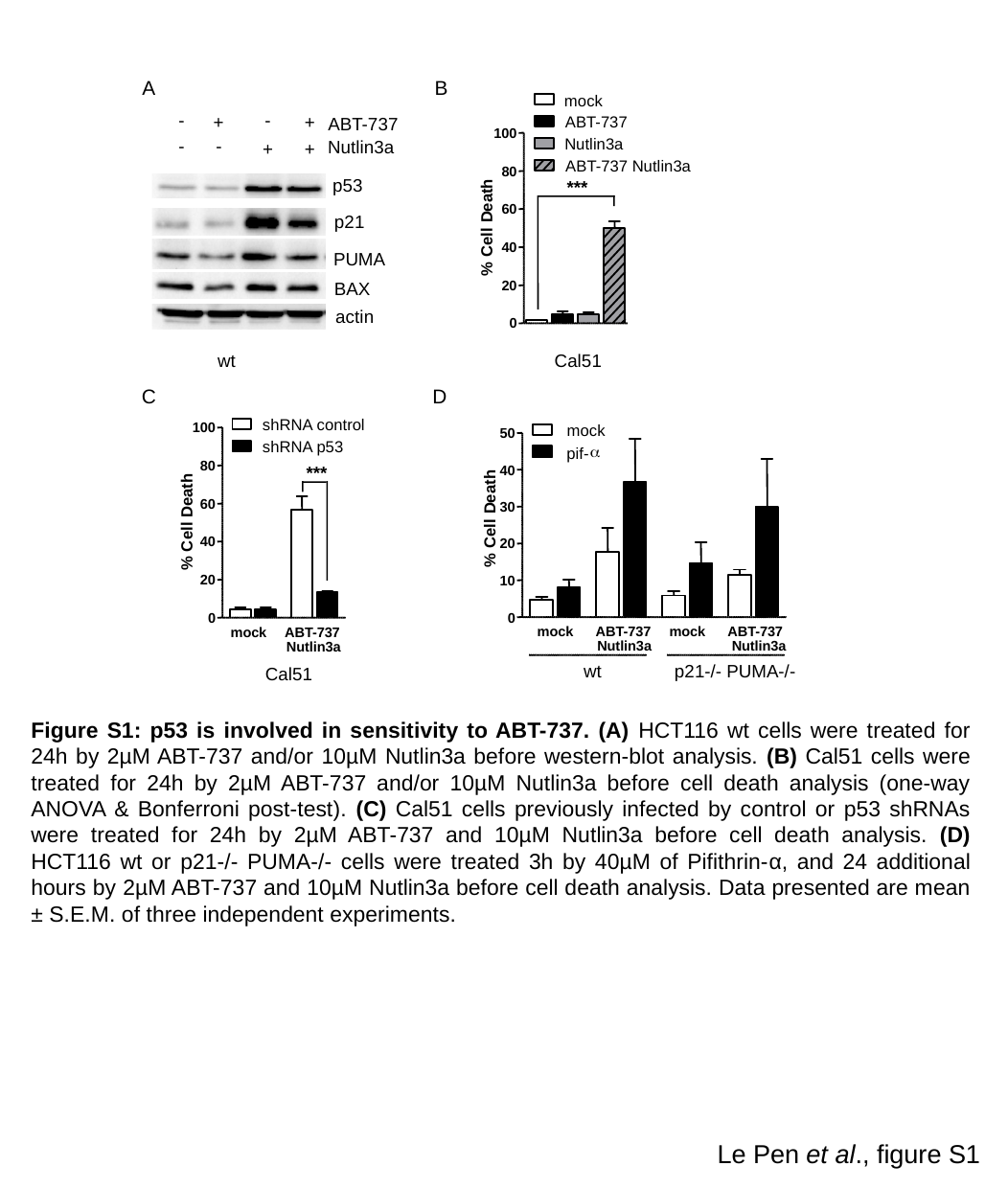

A
B
80
60
40
20
0
mock
ABT-737
100
Nutlin3a
ABT-737 Nutlin3a
% Cell Death
-
-
+
+
ABT-737
-
-
Nutlin3a
+
+
p53
***
p21
PUMA
BAX
actin
wt
Cal51
C
D
80
60
40
20
0
mock
ABT-737
shRNA control
100
shRNA p53
% Cell Death
Nutlin3a
Cal51
mock
ABT-737
mock
ABT-737
mock
50
α
pif-
40
30
20
10
0
Nutlin3a
wt
p21-/- PUMA-/-
% Cell Death
Nutlin3a
***
Figure S1: p53 is involved in sensitivity to ABT-737. (A) HCT116 wt cells were treated for 24h by 2µM ABT-737 and/or 10µM Nutlin3a before western-blot analysis. (B) Cal51 cells were treated for 24h by 2µM ABT-737 and/or 10µM Nutlin3a before cell death analysis (one-way ANOVA & Bonferroni post-test). (C) Cal51 cells previously infected by control or p53 shRNAs were treated for 24h by 2µM ABT-737 and 10µM Nutlin3a before cell death analysis. (D) HCT116 wt or p21-/- PUMA-/- cells were treated 3h by 40µM of Pifithrin-α, and 24 additional hours by 2µM ABT-737 and 10µM Nutlin3a before cell death analysis. Data presented are mean ± S.E.M. of three independent experiments.
Le Pen et al., figure S1

## Slide 2
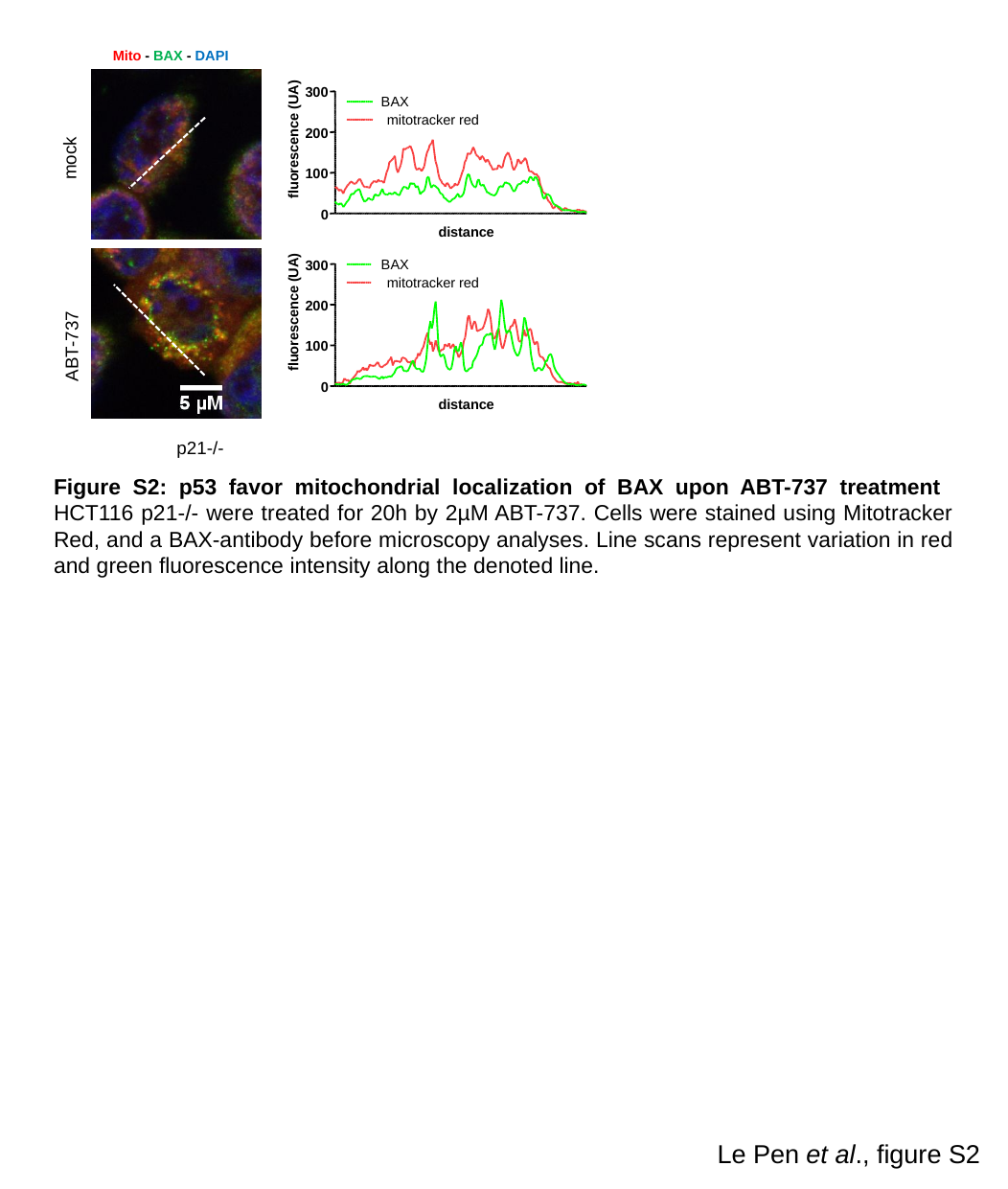

Mito - BAX - DAPI
300
200
100
0
BAX
mitotracker red
fluorescence (UA)
distance
mock
BAX
300
200
100
0
mitotracker red
fluorescence (UA)
distance
ABT-737
p21-/-
Figure S2: p53 favor mitochondrial localization of BAX upon ABT-737 treatment HCT116 p21-/- were treated for 20h by 2µM ABT-737. Cells were stained using Mitotracker Red, and a BAX-antibody before microscopy analyses. Line scans represent variation in red and green fluorescence intensity along the denoted line.
Le Pen et al., figure S2

## Slide 3
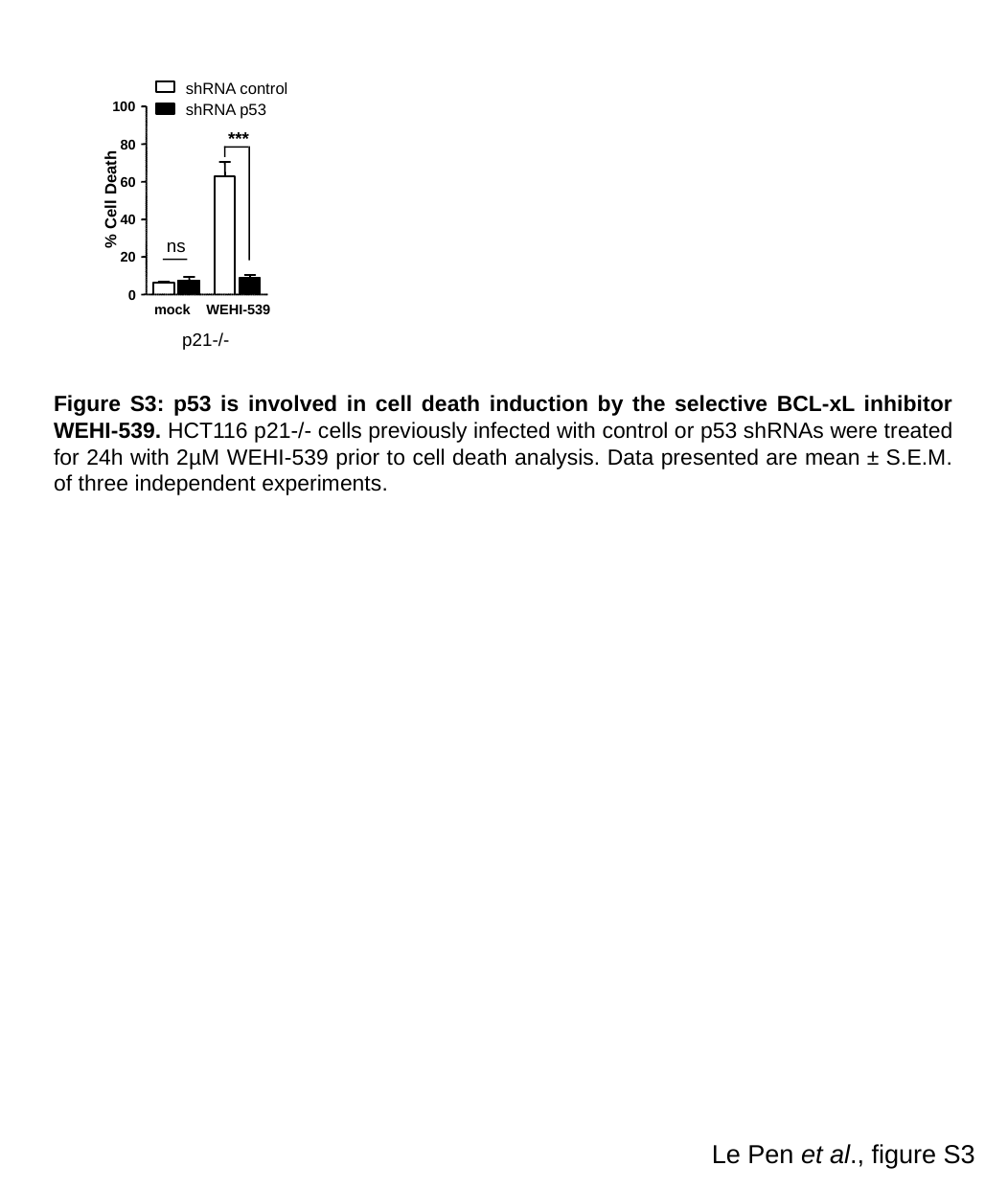

80
60
40
20
0
mock
WEHI-539
shRNA control
100
shRNA p53
% Cell Death
***
ns
p21-/-
Figure S3: p53 is involved in cell death induction by the selective BCL-xL inhibitor WEHI-539. HCT116 p21-/- cells previously infected with control or p53 shRNAs were treated for 24h with 2µM WEHI-539 prior to cell death analysis. Data presented are mean ± S.E.M. of three independent experiments.
Le Pen et al., figure S3

## Slide 4
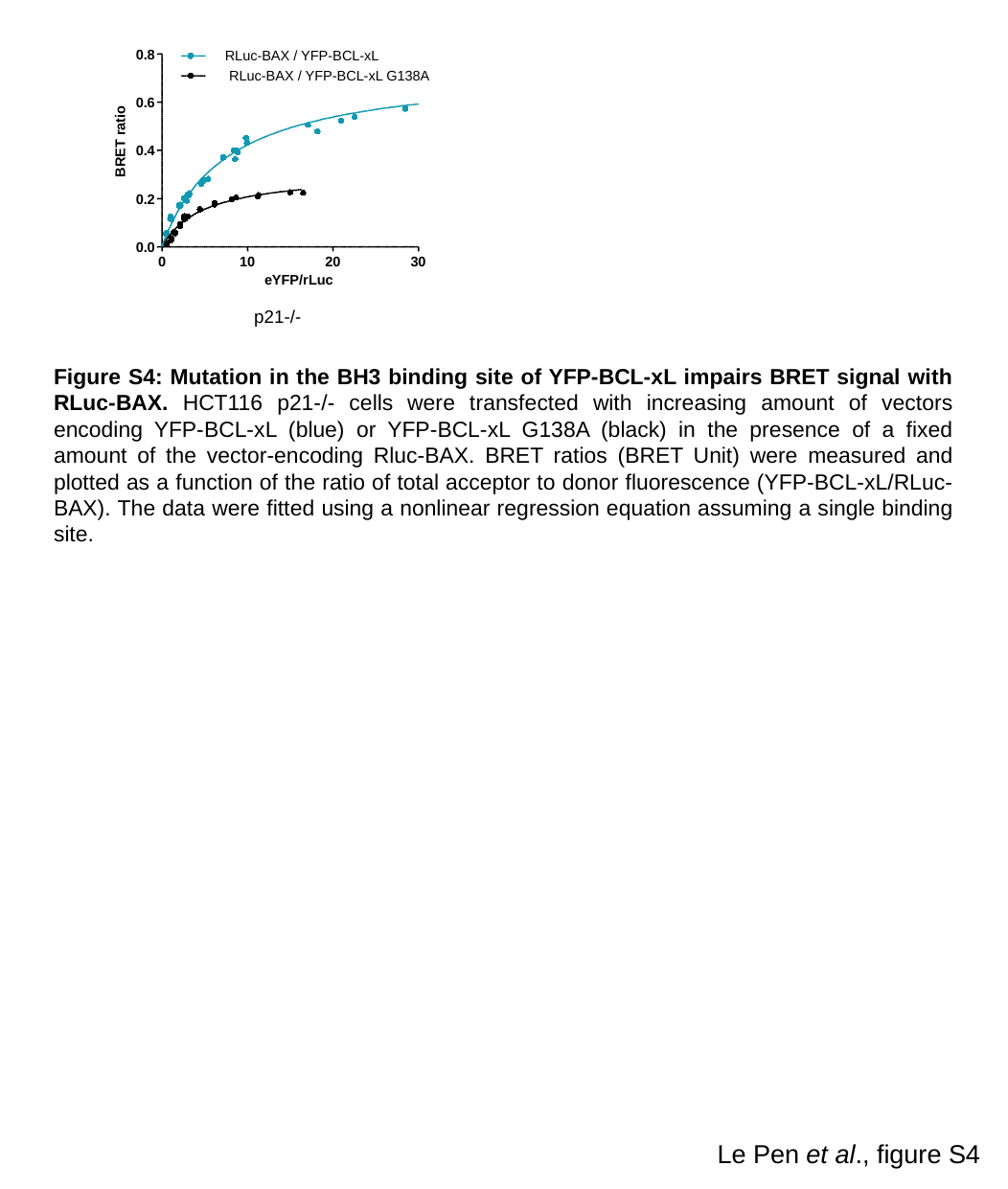

0
10
20
30
0.8
0.6
0.4
0.2
0.0
RLuc-BAX / YFP-BCL-xL
RLuc-BAX / YFP-BCL-xL G138A
BRET ratio
eYFP/rLuc
p21-/-
Figure S4: Mutation in the BH3 binding site of YFP-BCL-xL impairs BRET signal with RLuc-BAX. HCT116 p21-/- cells were transfected with increasing amount of vectors encoding YFP-BCL-xL (blue) or YFP-BCL-xL G138A (black) in the presence of a fixed amount of the vector-encoding Rluc-BAX. BRET ratios (BRET Unit) were measured and plotted as a function of the ratio of total acceptor to donor fluorescence (YFP-BCL-xL/RLuc-BAX). The data were fitted using a nonlinear regression equation assuming a single binding site.
Le Pen et al., figure S4

## Slide 5
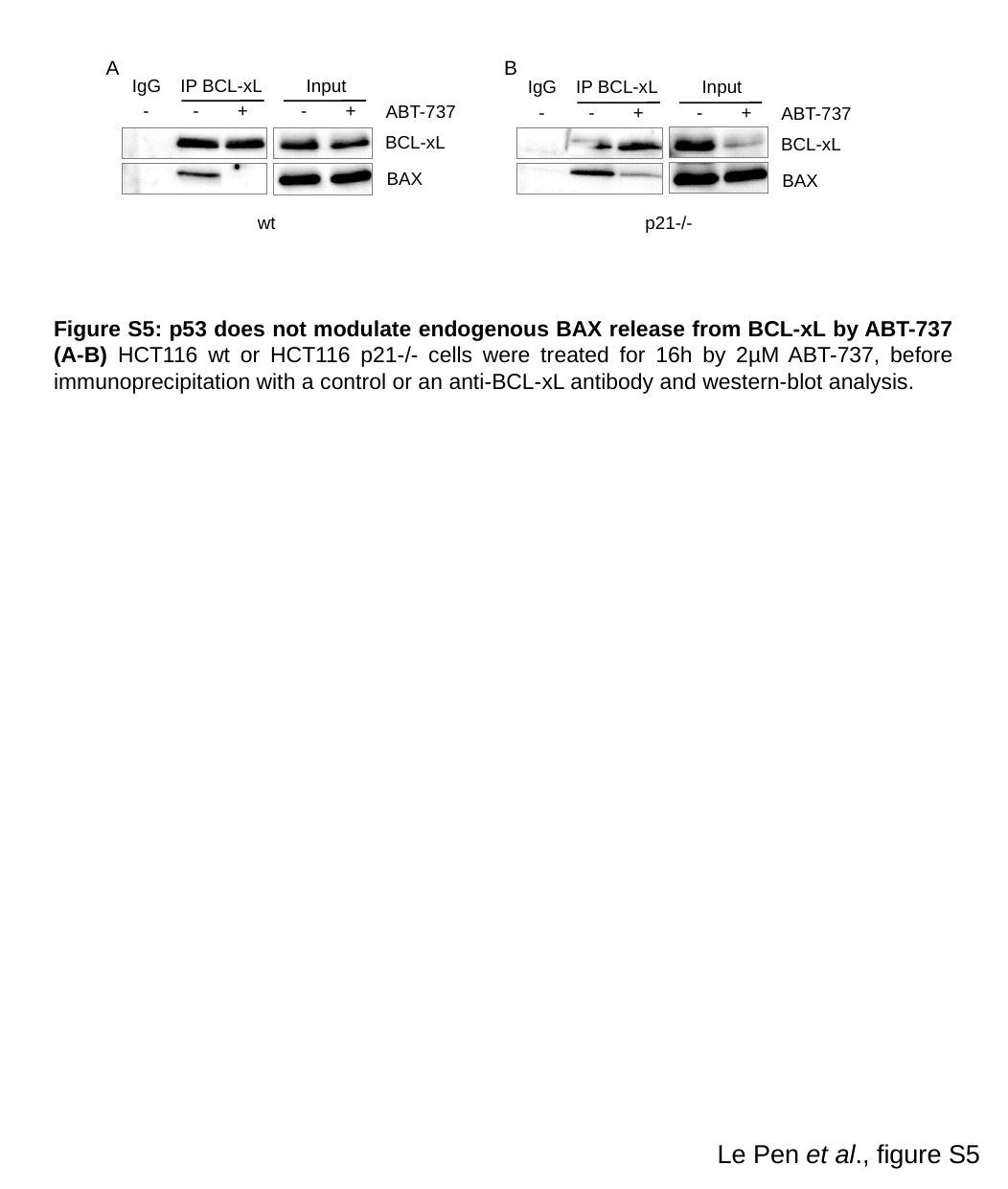

B
IgG
IP BCL-xL
Input
-
+
+
-
-
ABT-737
BCL-xL
BAX
A
IgG
IP BCL-xL
Input
-
+
+
-
-
ABT-737
BCL-xL
BAX
p21-/-
wt
Figure S5: p53 does not modulate endogenous BAX release from BCL-xL by ABT-737 (A-B) HCT116 wt or HCT116 p21-/- cells were treated for 16h by 2µM ABT-737, before immunoprecipitation with a control or an anti-BCL-xL antibody and western-blot analysis.
Le Pen et al., figure S5

## Slide 6
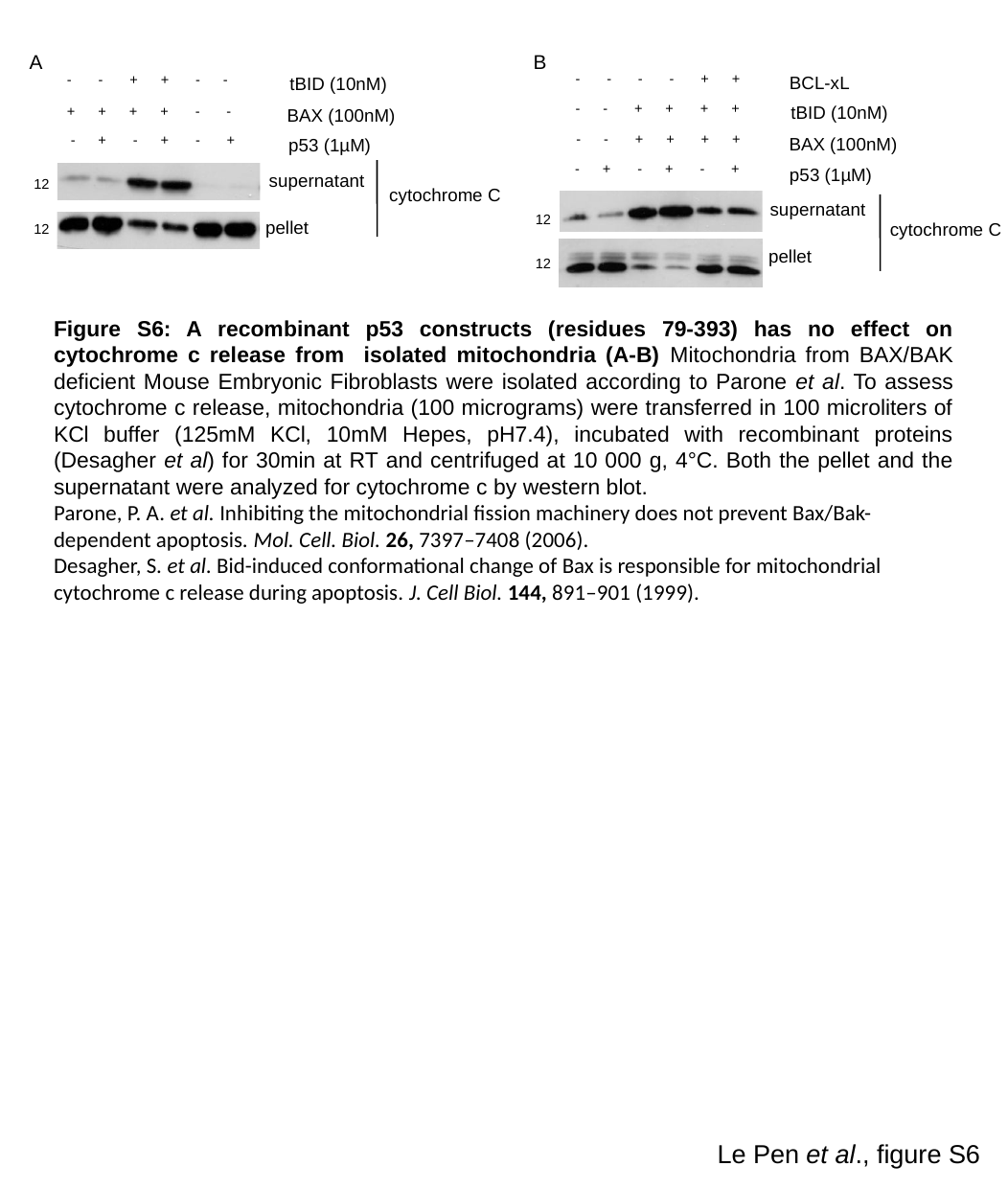

A
B
- - - - + +
- - + + - -
BCL-xL
tBID (10nM)
- - + + + +
tBID (10nM)
 + + + + - -
BAX (100nM)
 - - + + + +
- + - + - +
BAX (100nM)
p53 (1µM)
- + - + - +
p53 (1µM)
supernatant
12
cytochrome C
supernatant
12
pellet
cytochrome C
12
pellet
12
Figure S6: A recombinant p53 constructs (residues 79-393) has no effect on cytochrome c release from isolated mitochondria (A-B) Mitochondria from BAX/BAK deficient Mouse Embryonic Fibroblasts were isolated according to Parone et al. To assess cytochrome c release, mitochondria (100 micrograms) were transferred in 100 microliters of KCl buffer (125mM KCl, 10mM Hepes, pH7.4), incubated with recombinant proteins (Desagher et al) for 30min at RT and centrifuged at 10 000 g, 4°C. Both the pellet and the supernatant were analyzed for cytochrome c by western blot.
Parone, P. A. et al. Inhibiting the mitochondrial fission machinery does not prevent Bax/Bak-dependent apoptosis. Mol. Cell. Biol. 26, 7397–7408 (2006).
Desagher, S. et al. Bid-induced conformational change of Bax is responsible for mitochondrial cytochrome c release during apoptosis. J. Cell Biol. 144, 891–901 (1999).
Le Pen et al., figure S6
